# OsWRKY80-OsWRKY4 Module as a Positive Regulatory Circuit in Rice Resistance Against *Rhizoctonia solani*

**DOI:** 10.1186/s12284-016-0137-y

**Published:** 2016-11-25

**Authors:** Xixu Peng, Haihua Wang, Jyan-Chyun Jang, Ting Xiao, Huanhuan He, Dan Jiang, Xinke Tang

**Affiliations:** 1 School of Life Science, Hunan University of Science and Technology, Taoyuan Rd., Xiangtan, Hunan 411201 China; 2 Key Laboratory of Integrated Management of the Pests and Diseases on Horticultural Crops in Hunan Province, Xiangtan, Hunan 411201 China; 3 Key Laboratory of Ecological Remediation and Safe Utilization of Heavy Metal-polluted Soils, College of Hunan Province, Xiangtan, Hunan 411201 China; 4 Department of Horticulture and Crop Science, The Ohio State University, Columbus, OH 43210 USA; 5 Department of Molecular Genetics, The Ohio State University, Columbus, OH 43210 USA; 6 Center for Applied Plant Sciences, The Ohio State University, Columbus, OH 43210 USA

**Keywords:** Disease resistance, *Rhizoctonia solani*, Transcription factor, WRKY protein, *Oryza sativa*

## Abstract

**Background:**

Plant WRKY transcription factors play pivotal roles in diverse biological processes but most notably in plant defense response to pathogens. Sheath blight represents one of the predominant diseases in rice. However, our knowledge about the functions of WRKY proteins in rice defense against sheath blight is rather limited.

**Results:**

Here we demonstrate that the expression of *Oryza sativa WRKY80* gene (*OsWRKY80*) is rapidly and strongly induced upon infection of *Rhizoctonia solani*, the causal agent of rice sheath blight disease. *OsWRKY80* expression is also induced by exogenous jasmonic acid (JA) and ethylene (ET), but not by salicylic acid (SA). OsWRKY80-GFP is localized in the nuclei of onion epidermal cells in a transient expression assay. Consistently, OsWRKY80 exhibits transcriptional activation activity in a GAL4 assay in yeast cells. Overexpression of *OsWRKY80* in rice plants significantly enhanced disease resistance to *R. solani*, concomitant with elevated expression of *OsWRKY4*, another positive regulator in rice defense against *R. solani*. Suppression of *OsWRKY80* by RNA interference (RNAi), on the other hand, compromised disease resistance to *R. solani*. Results of yeast one-hybrid assay and transient expression assay in tobacco cells have revealed that OsWRKY80 specifically binds to the promoter regions of *OsWRKY4*, which contain W-box (TTGAC[C/T]) or W-box like (TGAC[C/T]) *cis*-elements.

**Conclusions:**

We propose that OsWRKY80 functions upstream of OsWRKY4 as an important positive regulatory circuit that is implicated in rice defense response to sheath blight pathogen *R. solani*.

**Electronic supplementary material:**

The online version of this article (doi:10.1186/s12284-016-0137-y) contains supplementary material, which is available to authorized users.

## Background

In the natural environment, plants are frequently confronted with diverse biotic and abiotic stresses that detrimentally affect their growth and development. Among them, pathogen attack is one of the most limiting factors of crop productivity and quality, and consequently poses a serious threat to agricultural industry worldwide. To ensure survival, plants have evolved intricate and robust mechanisms to respond to pathogen invasion through their innate immune system. Plant innate immune system is comprised of two interconnected branches. The first branch is pathogen-associated molecular pattern (PAMP)-triggered immunity (PTI), initiated by the recognition of molecular signatures of certain pathogens (e.g., bacterial flagellin and fungal chitin oligosaccharide). PTI often activates downstream mitogen-activated protein kinase (MPK) cascades and defense response genes. The second branch is effector-triggered immunity (ETI), which is a more accelerated defense response than PTI and is triggered by host-resistance (R) protein-mediated recognition of pathogen effectors (Jones and Dangl 2006). PTI- and ETI-mediated defense responses in plants are modulated mainly by three signaling hormone molecules, salicylic acid (SA), jasmonic acid (JA), and ethylene (ET) (Tsuda et al. 2009). There are both synergistic and antagonistic interactions between SA and JA/ET signaling pathways during plant immune progression (Kunkel and Brooks 2002; Mur et al. 2006). This apparent discrepancy reflects the complexity of plant defense mechanisms (Kim et al. 2006). Moreover, the expression of downstream defense-related genes is crucial for the establishment of plant immune responses. The interaction between plants and pathogens eventually leads to extensive transcriptional reprogramming of plant defense-responsive genes (Eulgem 2005), indicating that transcription factors play a pivotal role in plant disease resistance.

The transcription factor families involved in plant defense responses include TGA family of basic domain-leucine zipper (bZIP), ethylene response factor (ERF), MYB, WRKY and Whirly family proteins (Eulgem 2005). WRKY proteins are one of the largest families of transcription factors in plants with 72-74 members in Arabidopsis (Ülker and Somssich 2004; Dong et al. 2003) and over 100 members in rice (Wu et al. 2005; Ross et al. 2007). The WRKY factors are characterized by their conserved DNA-binding WRKY domains consisting of a highly conserved WRKYGQK stretch in N-termini, and a zinc-finger motif (C-X_4-5_-C-X_22-23_-H-X_1_-H or C-X_7_-C-X_23_-H-X_1_-C) in C-termini (Eulgem et al. 2000). The WRKY domain generally binds to the W-box (C/T)TGAC(C/T) or W-box like *cis*-elements in the promoters of target genes (Eulgem et al*.* 2000; Maleck et al. 2000). According to the number of WRKY domains and the features of zinc-finger motifs, the WRKY protein family is categorized into three distinct groups (I, II and III). Group II is further divided into five subgroups (IIa to IIe) based on the presence of additional short conserved structural motifs outside of the WRKY domain (Eulgem et al*.* 2000).

Loss- and gain-of-function studies have revealed that WRKYs act in a complex signaling network as both positive and negative regulators of various biological processes, but most notably in biotic stress responses (Pandey and Somssich 2009). To date, at least 13 *Oryza sativa* *WRKY* (*OsWRKY*) genes are known to positively regulate rice resistance against pathogens, such as *Magnaporthe oryzae*, *Rhizoctonia solani* and *Xanthomonas oryzae* pv *oryzae* (*Xoo*) (Cheng et al. 2015; Wang et al. 2015; Choi et al. 2015; Hwang et al. 2016). For instance, OsWRKY13 activates SA-dependent defense response whereas suppresses JA-dependent response, in mediating rice defense response to bacterial blight and fungal blast pathogens (Qiu et al. 2007). OsWRKY45 positively regulates systemic acquired resistance (SAR) in an SA-dependent manner (Shimono et al. 2007; Shimono et al. 2012). More recently, it has been reported that OsWRKY51 functions as a positive transcriptional regulator in defense signaling against *Xoo* by direct binding to the promoter of defense related gene, *OsPR10a* (Hwang et al. 2016). By contrast, although the transcription of *OsWRKY28*, 62, and *76* is upregulated upon pathogen infection, their protein products act to repress plant defense response against rice fungal blast or bacterial blight pathogens (Peng et al. 2008; Delteil et al. 2012; Chujo et al. 2013; Yokotani et al. 2013). More intriguingly, *OsWRKY45-1* derived from subspecies *japonica* acts as a negative regulator, whereas its allele *OsWRKY45-2* derived from subspecies *indica* as a positive regulator in the interactions between rice and bacterial pathogens such as *Xoo* and *X. oryzae* pv *oryzicola*. Nevertheless, both *OsWRKY45* alleles function as positive regulators in the defense against fungal pathogen *M. oryzae* (Tao et al. 2009). WRKYs often work in concert in plant defense response to pathogens. OsWRKY42 has been characterized as a negative regulator functioning downstream of OsWRKY13. OsWRKY42-OsWRKY13 together with OsWRKY45-2 form a WRKY transcriptional regulatory cascade in the rice- *M. oryzae* interaction (Cheng et al. 2015). The multiple roles of WRKYs suggest that the complex signaling and transcriptional networks of biotic stress responses require concerted regulation. Coordinated modulation of WRKY proteins as positive and negative regulators could also enable the proper amplitude and duration of plant response to minimize detrimental effects on plant growth and development during pathogen attack (Pandey and Somssich 2009).

Blast, caused by *M. oryzae*, bacterial blight, caused by *Xoo*, and sheath blight, caused by *R. solani* are considered to be three major diseases in rice. Among those characterized *OsWRKY* genes, at least 12 and 10 genes have been shown to function as either positive or negative regulators in rice resistance against *M. oryzae* and *Xoo*, respectively. However, only 2 *OsWRKY* genes (*OsWRKY30* and *OsWRKY4*) have been shown to mediate the defense responses against *R. solani* (Peng et al. 2012; Wang et al. 2015). Additionally, rice sheath blight is a necrotrophic disease (Zhao et al. 2008). The strategies of resistance to necrotrophs are distinct from those against biotrophs, and likely involved in defense mechanisms mediated by JA/ET-dependent signaling routes (Bari and Jones 2009). To elucidate the regulatory roles of rice WRKY factors in defense response to the sheath blight fungus, we have analyzed the expression profiles of rice *WRKY* family under *R. solani* infection and methyl jasmonate (MeJA) treatment. We have identified several pathogen- and JA-inducible *WRKY* genes, including *OsWRKY30* and *OsWRKY4*, which are positive regulators in rice resistance to *R. solani* (Peng et al. 2012; Wang et al. 2015). In this report, we investigate the expression pattern of *OsWRKY80* gene in response to exogenous defense-related phytohormones JA, ET and SA and *R. solani* challenge. We have found that OsWRKY80 is a nuclear-localized transcriptional activator. Compared to wild-type plants, the *OsWRKY80* overexpression rice plants are more resistant whereas knockdown (RNAi) lines are more susceptible to *R. solani* attack. In addition, we have found opposing expression pattern of *OsWRKY4* in gain- and loss-of function *OsWRKY80* plants, respectively*.* We have further demonstrated that OsWRKY80 specifically binds to the W-box, or W-box like *cis*-elements in the promoter of the *OsWRKY4* gene. Our findings suggest that OsWRKY80 functions upstream of OsWRKY4 and together this module acts as a positive regulatory circuit in the rice defense response against sheath blight disease.

## Results

### Cloning and Sequence Analysis of *OsWRKY80* cDNA

Several nomenclature systems of rice WRKY genes were proposed in the past by independent research groups (Zhang et al. 2004; Wu et al. 2005; Zhang and Wang 2005). To avoid the conflicts and confusion, the rice WRKY-working group has redefined the *WRKY* gene nomenclature based on the CGSNL (Committee on Gene Symbolization, Nomenclature and Linkage, Rice Genetics Cooperative) rules (Rice WRKY Working Group 2012). In the present study, we isolated a full-length cDNA of *OsWRKY80* gene. The *OsWRKY80* gene is located on chromosome 3 and designated with the locus number Loc_Os03g63810. This *OsWRKY80* is not to be confused with a previously reported *OsWRKY80* gene by Li et al. (2009) and Ricachenevsky et al. (2010), which is now designated as *OsWRKY90* (LOC _Os09g30400) according to the new CGSNL nomenclature. The obtained cDNA sequence of *OsWRKY80* was 1392 bp in length, containing an ORF of 1164 bp, encoding a polypeptide of 387 amino acid residues. Structure analysis revealed that the deduced OsWRKY80 consisted of one classic conserved WRKY domain with a zinc finger motif of C-X_5_-C-X_23_-H-X_1_-H, indicating that it belongs to the WRKY group II-e family (Eulgem et al*.* 2000). BLAST analysis revealed that OsWRKY80 shared the highest homology with previously uncharacterized OsWRKY37 (54.8%, LOC_Os04g50920) and AtWRKY14 (50.7%, At1g30650), respectively.

### Expression of *OsWRKY80* is Induced by JA, ET and *R. Solani*

**Fig. 1:**
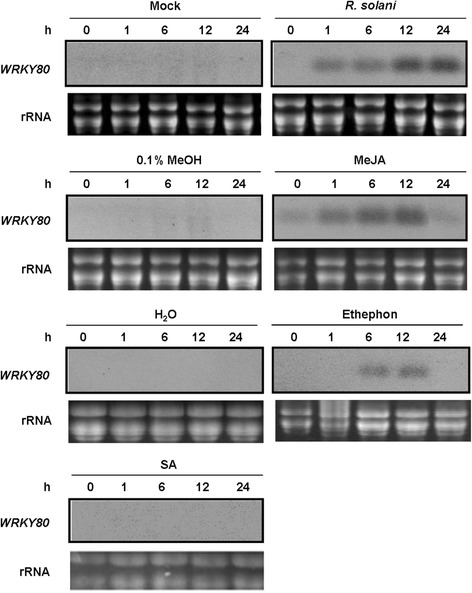
RNA gel blot analysis for expression of *OsWRKY80* in response to pathogen infection and chemicals. Total RNA was extracted from leaves of 3-week-old rice seedlings at the indicated time intervals after treatments. A 10 μg aliquot of total RNA was loaded per lane. The ethidium bromide stain of rRNA is shown for assessment of equal loading

WRKY transcription factors are frequently implicated in the regulation of plant immune responses (Pandey and Somssich 2009). To reveal if OsWRKY80 might be involved in the plant responses to biotic stresses, the expression of *OsWRKY80* was examined after the inoculation of *R. solani*. The *OsWRKY80* transcription was induced by *R. solani* at 1 h and peaked at 24 h (Fig. 1).

To determine the possible involvement of OsWRKY80 in hormone-mediated defense signaling pathways, we also monitored the expression of *OsWRKY80* following treatment with exogenously applied SA, JA and ET. As shown in Fig. 1, the expression of *OsWRKY80* was rapidly induced by JA within 1 h, peaked at 12 h, and sharply declined to basal levels at 24 h. The *OsWRKY80* transcripts were also noticeably induced by an ethylene precursor ethephon during 6–12 h before returning to basal levels at 24 h. However, exogenous SA application exerted no effects on the expression of *OsWRKY80*.

The strong induction of *OsWRKY80* expression by JA, ET and pathogen suggests that this gene may be involved in JA/ET-dependent defense signaling pathways.

### OsWRKY80 is Localized in the Nucleus

**Fig. 2:**
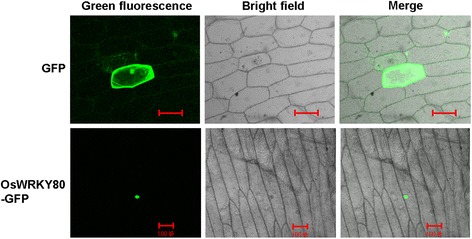
Nuclear localization of OsWRKY80. Onion epidermial cells were transformed with plasmids expressing GFP (top), or WRKY80-GFP fusion protein (bottom) and observed after 2 d under UV light (left panel) and white light (middle panel); Right panel is the merge of fluorescence and light. Bar = 100 μm

To determine the subcellular localization of the OsWRKY80 protein, we generated an *OsWRKY80-GFP* fusion gene under the control of the constitutive *CaMV 35S* promoter, and transiently expressed in onion epidermal cells via particle bombardment. As shown in Fig. 2, OsWRKY80-GFP was exclusively localized in the nucleus. By contrast, the GFP protein alone as a control was found throughout in the cytoplasm. The observation indicates that WRKY80 is a nuclear protein.

### OsWRKY80 Acts as a Transcriptional Activator in Yeast Cells

**Fig. 3:**
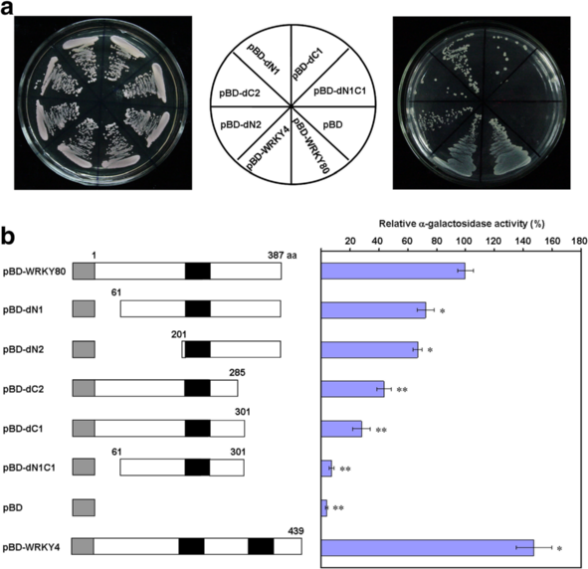
Transcriptional activation activity of OsWRKY80 in yeast cells. The full encoding sequence and deletion derivatives of *OsWRKY80* were fused in frame to the GAL4 binding domain (BD) in pBDKT7 (pBD) to generate various vectors for yeast transformation. **a** The constructed vectors were transformed into yeast AH109 strain, and grew on the selective medium at 30°C for 3 d. Yeast cells carrying different constructs grew on SD-Trp medium (left panel), or SD-Trp-Ade-His (right panel). Middle panel, schematic distribution of yeast cells carrying different vectors. **b** Assay for a-galactosidase activity. Empty vector pBD and pBD-WRKY4 (Wang et al, 2015) were used as negative and positive control, respectively. The enzymatic activity of cells carrying pBD-WRKY80 was set as 100%. Data are represented as mean values ± SE for three replicates. *, ** indicate a significant difference at *P* < 0.05 and 0.01, respectively, between the transformant for pBD-WRKY80 and other vectors according to Duncan’s multiple range test. Grey, black rectangles represent BD in pGBKT7 and WRKY domains, respectively. The numbers in each construct are the start and end positions of translation product of *OsWRKY80*

Based on the prediction using DNAStar software package, OsWRKY80 contains a C-terminal acidic region (*p*I = 4.0) with 6 consecutive glutamines (Q6) and 8 consecutive threonines (S8) that may function as a transcriptional activation domain (Triezenberg 1995; Schwechheimer and Bevan 1998). To determine if OsWRKY80 has transcriptional activation activity, we fused the full-length *OsWRKY80* in frame to the GAL4 DNA binding domain in the pGBKT7 vector and transformed into yeast strain AH109. Empty vector pBD and pBD-WRKY4 (Wang et al. 2015) were used as negative and positive control, respectively. The results showed that cells transformed with pBD-WRKY80 grew well on synthetic SD-Trp-Ade-His selection media (Fig. 3a), indicating that OsWRKY80 is a transcriptional activator in yeast cells. Next, to define the transcriptional activation domain of OsWRKY80, we generated a series of deletion constructs of *OsWRKY80* (pBD-dN1, -dN2, -dC1, -dC2 and -dN1C1), and conducted the same analysis. The results indicated that pBD-dN1 (deletion of 60 amino acid residues from the N-terminus), pBD-dC2 (deletion of 102 amino acid residues from the C-terminus) and pBD-dN1C1 (deletion of both N1 and C1) significantly reduced the transcription activity by 27.8% (*p* < 0.05), 72.1% and 92.9% (*p* < 0.01), respectively, as indicated by α-galactosidase activity assay. Interestingly, the α-galactosidase activity of pBD-dC1, which contains Q6 and S8 and is 25 aa longer than pBD-dC2, was significantly higher (*p* < 0.05) than that of pBD-dC2 (Fig. 3b), indicating that Q6 and S8 are important for the transcriptional activation activity. Together these findings suggest that both the N- and the C-terminal region are required for the full transcriptional activation activity of OsWRKY80.

### Modulation of Rice Resistance to Sheath Blight Fungus by OsWRKY80

**Fig. 4:**
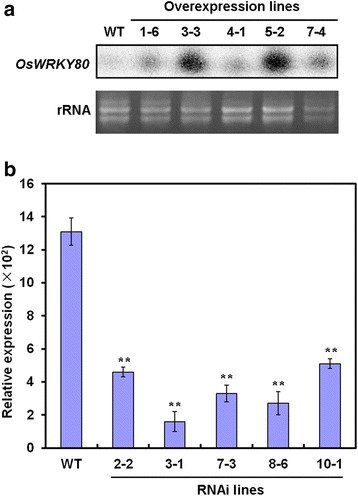
Analysis of the expression levels of *OsWRKY80* in transgenic rice lines under normal conditions. Total RNA was prepared from leaves of 4-week-old wild-type (WT) and T2 homozygous progeny of *OsWRKY80-*overexpressing and RNAi transgenic rice seedlings. **a** The expression of *OsWRKY80* in different overexpression lines was analyzed by RNA gel blot. A 10 μg aliquot of total RNA was loaded per lane. The ethidium bromide stain of rRNA is shown for assessment of equal loading. **b** The expression of *OsWRKY80* in different RNAi lines was determined by qRT-PCR analysis. The mRNA levels of *OsWRKY8*0 were calculated relative to those of rice *Actin1* (AK071586). Data are represented as mean values ± standard error (SE) for three replicates. ** indicates a significant difference at *P* < 0.01 between the transgenic and wild-type plants according to Duncan’s multiple range test

**Fig. 5:**
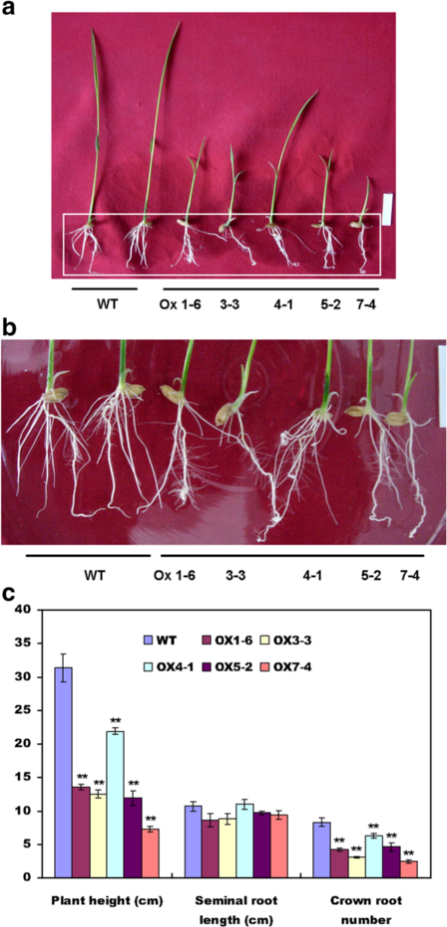
The phenotype of transgenic rice plants overexpressing *OsWRKY80*. **a** Plants (T2 generation) grew on ½ MS (containing 0.5% agar) for 14 d. Root architechure of each line in a box with white line is shown in (**b**). **c** Histogram showing plant height, seminal root length, crown root number of plants. At least 20 plants of each line were analyzed. Data are represented as mean values ± SE for three replicates. *, ** indicate a significant difference at *P* < 0.05, 0.01, respectively, between the transgenic and wild-type plants according to Duncan’s multiple range test. OX, overexpression lines; WT, wild-type plants

The induction of *OsWRKY80* expression in response to *R. solani* and exogenous JA and ET suggests its role for plant innate immunity. To test this possibility, we generated transgenic rice T2 lines ectopically expressing *OsWRKY80* driven by maize (*Zea mays*) *ubiquitin* promoter. RNA interference (RNAi) lines that express a 302-bp inverted-repeat sequence of *OsWRKY80* coding region were also generated. The construct for *OsWRKY80*-overexpression (OX) or RNAi was introduced into the rice cultivar Xiushui 11 by *Agrobacterium*-mediated transformation, and the expression of *OsWRKY80* in transgenic rice plants was determined by Northern blot and qRT-PCR analysis, respectively. Together, 10 and 12 independent transgenic lines of OX and RNAi, respectively, were obtained. Compared to those in the WT plants, *OsWRKY80* transcripts were evidently increased in different *OsWRKY80* OX lines (Fig. 4a). qRT-PCR revealed that the transcripts of *OsWRKY80* were significantly reduced in the RNAi lines (Fig. 4b). All *OsWRKY80* OX plants exhibited dwarfism and less crown roots compared with the WT plants (Fig. 5). However, no significant differences in the growth and morphology were observed between RNAi lines and the WT plants (data not shown).

**Fig. 6:**
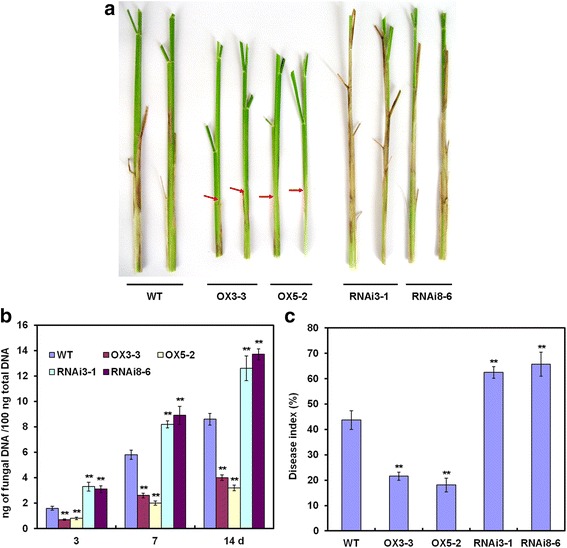
Resistance phenotypes of *OsWRKY80-*overexpressing and RNAi transgenic rice plants to *Rhizoctonia solani*. **a** Disease symptoms in wild-type (WT), *OsWRKY80*-overexpressing (lines OX3-3 and 5-2), and RNAi (lines 3-1 and 8-6) plants at 14 days after inoculation (DPI) with *Rhizoctonia solani* GD118. **b** Progression of sheath blight disease evaluated by quantitating *R. solani* genomic DNA using qPCR analysis. The amount of *R. solani* 28S rDNA was calculated relative to rice *RUBQ1* (AF184279) DNA. **c** Disease severity was evaluated as disease index at 14 DPI. Data are represented as mean values ± standard error (SE) for three replicates according to Duncan’s multiple range test (** *P* < 0.01). 20 plants for each genotype were used for each repetition

Next, we selected representative *OsWRKY80* OX (OX3-3 and 5-2) and RNAi lines (3-1 and 8-6) for further disease resistance tests. Four-week-old rice plants grown in greenhouse were inoculated with *R. solani* GD118 strain, and disease symptoms were evaluated 14 days post-inoculation (DPI). The results showed that *OsWRKY80* OX plants exhibited reduced susceptible lesions and disease severity compared with the WT plants (Fig. 6a, b). Consistently, the fungal growth in the OX lines was 55.2% (OX3-3) to 65.5% (OX5-2) lower than that in WT plants at 7 DPI (Fig. 6c). By contrast, the RNAi lines exhibited increased susceptibility to the fungal pathogen compared with the WT plants. These findings suggest that OsWRKY80 is a positive modulator of plant defense against the sheath blight fungus.

### OsWRKY80 Positively Regulates *OsWRKY4* Expression

**Fig. 7:**
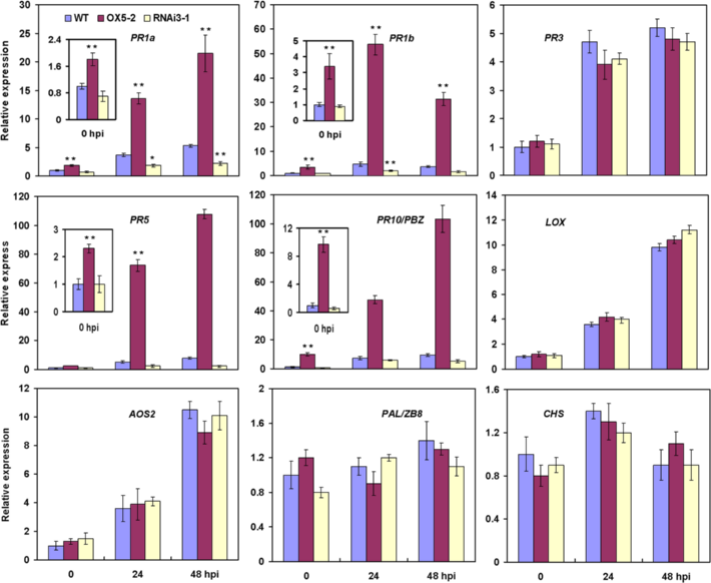
Changes in the expression of defense-related genes in *OsWRKY80-*overexpressing and RNAi transgenic rice plants. Four-week-old wild-type (WT), *OsWRKY80*-overexpression (line 5-2) and RNAi (line 3-1) plants were inoculated with *R. solani* race GD118, and RNA samples were generated from the fourth leaves of each genotype. The time course of gene expression was determined by qRT-PCR analysis. Transcription levels are expressed as the ratio to the level of transcript at 0 h in WT. Data are represented as mean values ± SE for three replicates. Asterisks indicate a significant difference between the transgenic plant and corresponding wild type within the same treatment (* *P* < 0.05, ** *P* < 0.01 according to Duncan’s multiple range test). hpi, hours postinoculation

Altered resistance phenotypes of OX and RNAi plants (Fig. 6) suggest that OsWRKY80 might control a subset of defense-related genes. To identify potential OsWRKY80 target genes, we evaluated the expression of several well-characterized defense-related genes in WT, *OsWRKY80-*OX and -RNAi plants. Interestingly, the results were highly similar to those obtained from the study of OsWRKY4 (Wang et al. 2015). The expression of *PR1a*, *PR1b*, *PR5* and *PR10/PBZ1* was elevated in the *OsWRKY80-*OX lines compared with the WT plants. In contrast, the expression of these marker genes was decreased in the *OsWRKY80* RNAi lines (Fig. 7). However, the transcript levels of *PR3*, *LOX*, *AOS2*, *PAL/ZB8* and *CHS* were not significantly different in the transgenic lines compared with the WT plants. These observations suggest that a subset of defense responsive genes is under the control of OsWRKY80 either directly or indirectly. It is noteworthy that these genes are also JA, or ET-responsive (Agrawal et al. 2001; Mei et al. 2006; Wang et al. 2015).

**Fig. 8:**
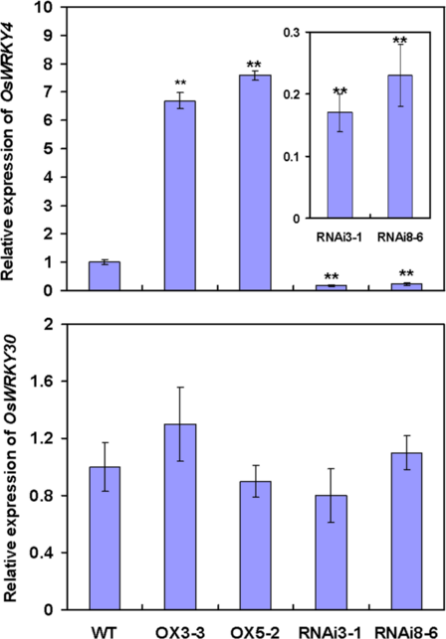
Expression of *OsWRKY4* and *30* in *OsWRKY80* transgenic rice lines by qRT-PCR analysis. RNA samples were prepared from leaves of 4-week-old wild-type (WT) and T2 homozygous progeny of *OsWRKY80*-overexpressing and RNAi transgenic rice seedlings. Expression was normalized to that of rice *Actin1*(AK071586). The transcript level from the wild-type plants was set to 1. Data are represented as mean values ± SE for three replicates. ** indicates a significant difference at *P* < 0.01 between the transgenic and wild-type plants according to Duncan’s multiple range test

*OsWRKY80* and *OsWRKY4* display similar expression patterns in response to JA, ET and *R. solani* (Fig. 1; Wang et al. 2015). Moreover, they regulate the expression of the same subsets of JA-, or ET-responsive defense-associated genes (Fig. 7). These findings raise a possibility that OsWRKY4 and OsWRKY80 coordinately modulate rice defense response to *R. solani* possibly via a JA/ET signaling pathway. To test this hypothesis, we evaluated in *OsWRKY80-*OX and -RNAi lines the expression of *OsWRKY4* and *OsWRKY30* genes, which have been previously characterized as postive modulators in rice defense response to *R. solani* (Peng et al. 2012; Wang et al. 2015). Only *OsWRKY4* was upregulated in the *OsWRKY80-*OX lines and downregulated in the *OsWRKY80-*RNAi lines (Fig. 8). However, no changes in *OsWRKY80* expression were observed in *OsWRKY4* transgenic plants. Likewise, the expression levels of *OsWRKY80* and *OsWRKY30* in the *OsWRKY4* transgenic lines, or those of *OsWRKY80* and *OsWRKY4* in the *OsWRKY30* transgenic lines were not significantly changed (data not shown). These findings suggest that OsWRKY4 acts downstream of OsWRKY80, and OsWRKY30 may act independently of OsWRKY80 and OsWRKY4 in the defense response pathway against rice sheath blight.

**Fig. 9:**
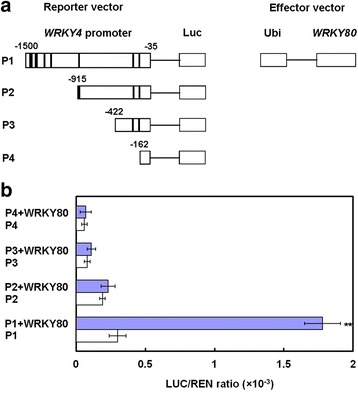
Transient assay for the interaction between OsWRKY80 and the promoter of *OsWRKY4* in tobacco. **a** Diagram of the promoter of *WRKY4* showing W-box or W-box like elements (bold vertical lines) in different regions. **b** Transient expression assay in tobacco. Full-length (P1) and 5’-deleted (P2, P3 and P4) promoters of *OsWRKY4* were constructed into the report vector, and *OsWRKY80* was cloned into the effect vector, respectively. LUC, Firefly luciferase activity; REN, Renilla luciferase activity (used as the control). Data are represented as mean values ± SE for three replicates. Asterisks indicate a significant difference (** *P* < 0.01) between the co-infiltration with reporter and effector vectors and the infiltration with reporter vectors alone according to Duncan’s multiple range test

**Fig. 10:**
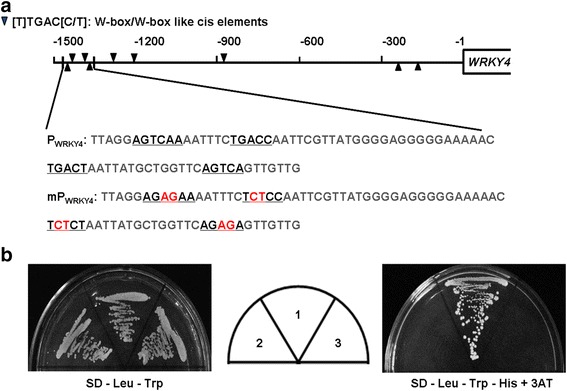
OsWRKY80 binds to *OsWRKY4* promoter in yeast. **a** Distribution of W-box and W-box like elements in the *OsWRKY4* promoter, 1.5 Kb upstream from the translational start site. Black triangles indicate W-box or W-box like elements. A 79-nucleotide-long promoter fragment, which contains four W-box or W-box like elements, was indicated, and used for DNA-binding test in yeast. The W-box or W-box like elements are underlined and in black. Bases in red were indicated as the mutant sites. **b** Yeast cells were co-transformed with a bait vector, containing a promoter fragment in (**a**) fused to a *HIS2* reporter gene, and a prey vector, containing OsWRKY80 fused to a GAL4 activation domain. Left, yeast cells carrying different constructs were grown for 2 d at 30°C in SD-Leu-Trp agar medium. Middle, schematic distribution of yeast cells carrying different vectors; 1, OsWRKY80 + P*WRKY4*; 2, OsWRKY80 + mP*WRKY4*; 3, P*WRKY4* only. Right panel, positive interactions between OsWRKY80 and target DNA fragments were verified in SD-Leu-Trp-His agar medium with 30 mM 3-amino-1, 2, 4-triazole (3-AT) for suppression of background growth

WRKY proteins specifically bind the W-boxes or W-box like elements containing the TGAC core sequence which often exist in the promoters of many defense-related genes, including *WRKY* genes themselves (Eulgem et al*.* 2000; Maleck et al. 2000; Turck et al. 2004). Promoter analysis using the PLACE database (http://tenor.dna.affrc.go.jp/) revealed that 2 W-box and 7 W-box like sequences were distributed in the 1.5 kb-promoter of *OsWRKY4* (Wang et al. 2015; Fig. 10a). To test whether OsWRKY80 specifically binds to W-box *cis*-elements in the promoter of *OsWRKY4* in planta, we conducted a transient expression assay by agro-infiltration of *Nicotiana benthamiana* leaves (Yang et al. 2000). The full (P1) and 5’-trucated (P2, P3 and P4) promoters of the *OsWRKY4* gene were cloned into a reporter vector with dual luciferases, and infiltrated alone or together with a modified pCambia1301 in which *OsWRKY80* is expressed under the control of a maize *ubiquitin* promoter (Fig. 9a). Results showed that OsWRKY80 significantly enhanced reporter activity driven by the intact promoter (P1) of *OsWRKY4*, but not by the 5’-deleted P2, P3 and P4 fragments (Fig. 9b). These findings indicate that OsWRKY80 can bind to the promoter of *OsWRKY4* in plant cells, and the deleted sequence between P1 and P2 fragments plays a key role in OsWRKY80-mediated *OsWRKY4* expression.

Furthmore, we used the 5’ region (-1429-1356 bp) of *OsWRKY*4 promoter, which contains 4 tandem W-box or W-box like elements, and the same region with mutations as baits and performed yeast one-hybrid assays. The interactions between OsWRKY80 and these promoter fragments were determined by yeast growth on agar media (-Trp, -Leu, -His) supplemented with 30 mM 3-AT for suppression of leaky growth. As shown in Fig. 10b, cells cotransformed with OsWRKY80 and the native *OsWRKY4* promoter fragments grew well on the selective media, whereas those with the corresponding mutant promoter fragments could not grow. These results again indicate that OsWRKY80 could specifically bind to the promoter of *OsWRKY4*. Interestingly, additional yeast one-hybrid analysis revealed that OsWRKY80 could not bind its own promoter in yeast cells (data not shown), suggesting that OsWRKY80 does not regulate its own expression.

## Discussion

Emerging evidence has highlighted the importance of WRKY factors as positive or negative regulators in rice disease resistance networks (Pandey and Somssich 2009). Global gene expression profiling has revealed a large number of *WRKY* genes that are rapidly induced or repressed upon pathogen infection, suggesting that these *WRKY* genes may contribute to the regulation of rice response to pathogen infection (Ryu et al. 2006; Bagnaresi et al. 2012; Wei et al. 2013). Moreover, some rice WRKY factors have been characterized to be involved in plant defense responses (Pandey and Somssich 2009; Jimmy and Babu 2015; Phukan et al. 2016). However, the roles of WRKY transcription factors in rice denfense against sheath blight is quite limited. Overexpression of *OsWRKY30* enhanced resistance to *R. solani* and *M. oryzae* possibly by activating several downstream genes, including JA biosynthesis-related genes (Peng et al. 2012). OsWRKY4 acts as a transcriptional activator in modulating defense response against *R. solani* (Wang et al. 2015). Both OsWRKY4 and OsWRKY30 belong to group I family. Here, we have added a novel *OsWRKY80* gene to the list of WRKY defense regulators in rice- *R. solani* interaction. Our results demonstrate that overexpression of *OsWRKY80* significantly enhanced, whereas suppression of *OsWRKY80* by RNAi markedly compromised sheath blight resistance. Furthermore, we show that OsWRKY80 acts upstream of the previously characterized OsWRKY4 in defense signaling pathway against *R. solani*. On the basis of these results, we conclude that OsWRKY80-OsWRKY4 regulatory circuit plays a positive role in rice defense response to sheath blight infection.

Compared to other groups, a higher percentage of rice WRKY Group II proteins are involved in plant defense responses to pathogens. A systematic expression analysis of *OsWRKY* genes revealed that the expression of one-third of tested genes was upregulated in response to an incompatible interaction between rice and *M. grisea*. Among the inducible *OsWRKY* genes, 8 genes belong to the members of Group II subfamily (Ryu et al. 2006). Moreover, 10 out of 16 *OsWRKY* genes, which have been characterized to mediate plant innate or induced immunity in rice, belong to Group II. For instance, OsWRKY13 positively mediates rice defense responses against both *Xoo* and *M. grisea* (Qiu et al. 2007). OsWRKY6, 13/03, 51 and 71 also positively mediate rice resistance to *Xoo* (Choi et al. 2015; Liu et al. 2005; Hwang et al. 2016; Liu et al. 2007). On the other hand, some Group IIa proteins, such as OsWRKY28, 62 and 76 are transcriptional repressors, and they act as negative regulators in rice response to *M. grisea* or *Xoo* (Chujo et al. 2013; Peng et al. 2008; Yokotani et al. 2013). In the present study, OsWRKY80, a member of the IIe subgroup protein, has also been identified as a positive modulator of rice resistance to sheath blight. Together these results suggest that Group II WRKY proteins represent a major force in mediating plant defense responses against various pathogens. In support of this notion, Arabidopsis Group IIe AtWRKY22 and 29 have been shown as important downstream components of a MAPK pathway that confers resistance to both bacterial and fungal pathogens (Asai et al. 2002). Conversely, AtWRKY27 negatively modulates symptom development caused by *Ralstonia solanacearum* infection (Mukhtar et al. 2008).

To date, only a few investigations have revealed regulatory cascades amongst different WRKYs. OsWRKY45-2, functioning as a transcriptional activator, directly activates WRKY13. OsWRKY13 functioning as a transcriptional repressor, in turn suppresses OsWRKY42. The three WRKYs form a transcriptional regulatory cascade in the defense signaling pathway against *M. oryzae*. In the present study, we have identified a new OsWRKY80-OsWRKY4 regulatory circuit in the rice defense signaling pathway to *R. solani*. First, OsWRKY80 and OsWRKY4 control the same subset of defense-related genes such as *PR1a*, *PR1b*, *PR5* and *PR10/PBZ1*, suggesting that the two regulators may be located in the same pathway. Second, the transcript levels of *OsWRKY4* were activated by *OsWRKY80-*overexpression, whereas suppressed by *OsWRKY80* RNAi, suggesting that *OsWRKY4* may be a target gene of OsWRKY80. By contrast, no changes in the expression of *OsWRKY80* were observed in *OsWRKY4*-overexpressing or -RNAi transgenic rice plants. Finally, the promoter region of the *OsWRKY4* gene contains 9 W-box or W-box like elements. Yeast one-hybrid assay and transient expression analysis in tobacco cells results showed that OsWRKY80 could specifically bind to the *OsWRKY4* promoter, and the 5’ promoter region containing W-box, or W-box like elements is responsible for the binding activity. Together these lines of evidence clearly suggest that OsWRKY80 acts upstream of OsWRKY4 by regulating its expression.

Functional studies have suggested an intricate regulatory network involved in both WRKY transcription factors and phytohormone signaling pathways. WRKY proteins frequently act as key components and interact with diverse partners related to hormone signaling pathways (Jiang and Yu 2015). In plants, pathogen attack often triggers multiple defense-response signaling pathways mediated by SA, JA and ET. Multiple lines of evidence have also revealed the roles of WRKY factors in modulating the balance between SA- and JA/ET-mediated signaling pathways. For instance, AtWRKY33, a positive regulator of JA/ET-mediated defense response signaling and a negative regulator of SA-mediated defense response signaling, plays an important role in plant defense against necrotrophic pathogens (Zheng et al. 2006). By contrast, OsWRKY13 appears to promote SA-dependent and suppress JA-dependent defense responses, acting in a convergent point of the two defense signal pathways (Qiu et al. 2007). Recently, we have identified OsWRKY4 as a crucial positive regulator in JA/ET-mediated defense signaling pathway (Wang et al. 2015). In this study, *OsWRKY80* and *WRKY4* gene exhibited similar expression pattern induced by JA, ET and *R. solani*, bu not by SA (Fig. 1; Wang et al. 2015). More importantly, OsWRKY80 directly binds the promoter of the *OsWRKY4* gene, suggesting that OsWRKY4 acts downstream of OsWRKY80 in the defense signaling pathway. These findings strongly suggest that OsWRKY80 may affect defense responses through JA/ET-mediated signaling pathway.

SA and JA/ET signaling pathways mediate resistance against different types of microbial pathogens. SA is usually involved in resistance against biotrophic pathogens (Hammond-Kosack and Parker 2003). On the other hand, the synergistic action of JA and ET is usually induced by necrotrophic pathogens and insects (Bari and Jones 2009). AtWRKY33 is a positive regulator of JA responses. Ectopic overexpression of *AtWRKY33* increases resistance to necrotrophic fungal pathogens *Botrytis cinerea* and *Alternaria brassicicola*, concomitant with reduced expression of JA-regulated gene *PDF1.2* (Zheng et al. 2006). Shimono et al. (2007) reported that overexpression of *OsWRKY45*, a positive regulator in BTH-induced disease resistance by mediating SA signaling, enhanced resistance against hemibiotrophic *M. oryzae* and biotrophic *Xoo*, but not against necrotic *R. solani* (Shimono et al. 2012). The results presented here showed that OsWRKY80 plays a positive role in the resistance to a necrotic fungal pathogen (Fig. 5). These findings are consistent with the facts that OsWRKY80 activates *OsWRKY4* expression, and subsequently activates JA/ET-dependent defense responses, thus protecting rice plants from the necrotic fungus. Thus, both dicots and monocots may share similar defense mechanisms to diverse pathogens through discrete phytohormone-mediated signal pathways.

## Conclusions

This study provides data on the role of OsWRKY80 in defense responses. Our results clearly demonstrated that OsWRKY80 contributes to activating defense responses to the rice sheath blight fungus by directly controlling OsWRKY4 via JA/ET-mediated signal pathway. To gain more insights into the integral regulatory network mediated by OsWRKY80 and OsWRKY4, further investigations are needed to explore additional functional linkages with MPK cascades and other novel components.

## Methods

### Plant Material, Growth Conditions and Chemical Treatments

Rice plants (*Oryza sativa* L. japonica cv. Xiushui 11) were grown in a greenhouse with day/night cycle of 14/10 h, 28°C/25°C, photosynthetically active radiation (PAR) of 100 μmol m^-2^ s^-1^, and relative humidity around 85% in hydroponic culture, as previously described by Peng et al. (2012).

Three-week-old rice seedlings were sprayed with 1 mM SA, 100 μM MeJA and 1 mM ethephon (an ethylene generator) until liquid dripped off the leaves. Control plants were treated in the same way with diluted water or 0.1% methanol (for MeJA only). Leaf samples were harvested at given time points after treatments for total RNA extraction.

### Pathogen Inoculation and Disease Investigation

Inoculation with *R. solani* (race GD118) was carried out according to Wang et al. (2009). Sheath blight disease progression was quantified by measurement of fungal genomic 28S rDNA relative to a rice *RUBQ1* gene (AF184279) (Wang et al. 2000) using quantitative real-time PCR (qRT-PCR) analysis (Sayler and Yang 2007). The primer sequences are listed in Additional file 1: Table S1. Symptom development was observed after 14 d and rated on a 0–9 scale based on leaf area affected (Rush et al. 1976). The disease index was calculated by using the formula:

$$ \begin{array}{l}\mathrm{Disease}\ \mathrm{in}\mathrm{d}\mathrm{ex} = \Big[\varSigma \left(\mathrm{The}\ \mathrm{number}\ \mathrm{of}\ \mathrm{d}\mathrm{isease}\mathrm{d}\ \mathrm{plants}\ \mathrm{in}\ \mathrm{this}\ \mathrm{scale}\kern0.5em \times \kern0.5em \mathrm{Disease}\ \mathrm{scale}\right)/\Big(\mathrm{Total}\ \mathrm{number}\ \mathrm{of}\ \mathrm{plants}\\ {}\ \mathrm{in}\mathrm{vestigated}\kern0.5em \times \kern0.5em \mathrm{The}\ \mathrm{highest}\ \mathrm{d}\mathrm{isease}\ \mathrm{scale}\left)\right]\kern0.5em \times \kern0.5em 100\%.\end{array} $$

### Isolation of Os*WRKY80* cDNA

The full-length cDNA of *OsWRKY80* was amplified from the total RNA extracted from MeJA-infected rice seedlings by RT-PCR using specific primer pairs: forward primer, 5’- AATACTGAATAGGCAGCAGCAACA -3’ and reverse primer, 5’- GCACAGGCGACCATCATATCATAT -3’. The primers were designed according to the annotated *OsWRKY80* gene (Loc_Os03g63810) including its longest open reading frame (ORF). The PCR products were cloned to a pUCmT vector and sequenced for verification.

### Gene Expression Analysis

Total RNA was extracted using PureYield™ RNA Midiprep System (Promega). Reverse transcription was performed using 2 μg of total RNA treated with DNase I (Invitrogen) and SuperScript reverse transcriptase II (Invitrogen) according to the manual. qRT-PCR was performed as previously described (Peng et al. 2011). Each experiment was repeated independently three times. Rice *Actin1* (AK071586) was used as internal reference (Qiu et al. 2008). Northern blot analysis was carried out as described by Sambrook et al. (1989). The GenBank accession numbers of the defense-related genes examined in the qRT-PCR analysis are as follows: *PR1a* (AJ278436), *PR1b* (AK107926), *PR3* (D16221), *PR5* (OSU77657), *PR10/PBZ1* (D38170), *LOX* (D14000), *AOS2* (AY062258), *CHS* (NM_001058538) and *PAL/ZB8* (KF556681). The gene-specific primers for gene expression analysis are listed in Additional file 1: Table S1.

### Subcellular Localization

The coding sequence of *OsWRKY80* was fused in frame to the N-terminus of an enhanced green fluorescent protein gene (*eGFP*) in p35S: eGFP (Wang et al. 2007) to generate p35S: WRKY80-eGFP construct. The primers are as follows: 5’-TTGGATCC ATGGATATGATGGAGGAGGA-3’ and 5’-TCACTCGAGGAACTTGTGCCACTGATGATCA-3’ with an underlined BamH I site and Sal I site, respectively. The empty p35S: eGFP vector was used as control. The fusion and the control constructs were transformed into onion (*Allium cepa*) epidermis cells by particle bombardment using PDS-1000/He (BIO-RAD) (Xie et al. 2003). The transformed cells were incubated on 1/2 MS medium at 28°C for 2 d, and GFP signals were detected by a confocal fluorescence microscope (Bio-Rad MRC 1024).

### Transactivation Activity Assay in Yeast

The coding region of *OsWRKY80* and its truncated fragments were amplified by PCR and fused to the GAL4 DNA binding-domain (BD) vector pGBKT7 (Clontech) to generate pBD-WRKY80, -dN1 (61-387), -dN2 (201-387), -dC1 (1-301), -dC2 (1-285) and -dN1C1 (61-301) constructs. The PCR primers are listed in Additional file 2: Table S2. The empty vector pGBKT7 was used as negative control. The yeast strain AH109 was transformed with different constructs and grown on SD-Trp-Ade-His selective medium at 30°C for 3 d. The α-galactosidase activity assay was performed with the transformed cell lines grown in liquid SD-Trp medium using *p*-nitrophenyl α-D-galactopyranoside as a substrate according to the manual.

### Generation of Transgenic Plants

The full-length coding sequence of *OsWRKY80* was digested with BamH I and Sac I and inserted into a modified pCambia1301 vector under the control of the constitutive maize (*Zea mays*) *ubiquitin* promoter (Wang et al. 2007). To construct a plasmid for OsWRKY80 RNAi, part of the WRKY80 cDNA (302 bp, nucleotides 775 to 1076) was amplified by PCR and used to construct self-complementary hairpin vector pCo-Ubi: dsWRKY80 after several steps of enzyme digestions and ligations. The hairpin structure, which is composed of the sense and antisense of *OsWRKY80* cDNA fragments seperated by a catalase intron, was put under the control of constitutive maize *ubiquitin* promoter. The resulting constructs were introduced into rice calli of cultivar Xiushui 11 by the *Agrobacterium*-mediated transformation method (Hiei et al. 1994).

### Promoter Fragments-Binding in Yeast one-Hybrid System

For analysis of the putative W-box (TTGAC[C/T]) or W-box like (TGAC[C/T]) *cis*-elements in promoters, the 1500 nucleotide sequences upstream of the transcription initiation sites of genes were used to search the PLACE (Plant *Cis*-acting Regulatory DNA Elements) database available online (http://tenor.dna.affrc.go.jp/).

*OsWRKY80* coding region was amplified and in frame fused with the GAL4 activation domain of pGADT7-rec2 prey vector (Clontech), forming pGAD-WRKY80. Construction of pHIS2 vector, yeast cotransformation and growth were performed as previously described (Wang et al. 2015).

### Transient Expression Assay in Tobacco (*Nicotiana Benthamiana*)

The construct for generation of transgenic plants described above was used as the effector vector for transient expression assay. The intact (about 1500 bp) and 5′-deleted promoters of *OsWRKY4* were amplified and constructed into the reporter vector pGreenII0800-LUC (Hellens et al. 2005). The recombinant reporter and effector plasmids, or reporter plasmids alone, were transferred into the *Agrobacterium* GV3101 lines, and infiltrated into the *N. benthamiana* leaves as described previously by Yang et al. (2000). The Firefly and Renilla luciferase activities were measured using a Dual Luciferase assay kit (Promega) according to the manufacturer’s instructions. The primers used are listed in Additional file 3: Table S3.

### Statistical Analysis

Data are represented as mean values ± standard error (SE) for three replicates. Analysis of variance (one-way ANOVA) and multiple comparisons of differences between treatments (Duncan’s multiple range test, *p* < 0.05, 0.01) were performed using SPSS for Windows version 11.5 (SPSS Inc.).

## Additional files

Additional file 1: Table S1. Gene-specific primers for quantitative real-time PCR or Northern blot analysis. (DOC 48 kb)
Additional file 2: Table S2. Specific primers of transcriptional activity anlysis in yeast cells. (DOC 34 kb)
Additional file 3: Table S3. Specific primers for amplification of the full and 5’-deleted promoters of *OsWRKY80. (DOC 29 kb)*
