# Supplementary material for: OsWRKY80-OsWRKY4 Module as a Positive Regulatory Circuit in Rice Resistance Against Rhizoctonia solani
Source: Rice (N Y). 2016 Nov 25;9:63. doi: 10.1186/s12284-016-0137-y (PMC5124021; doi:10.1186/s12284-016-0137-y)
Supplement: Additional file 1: Table S1. — Gene-specific primers for quantitative real-time PCR or Northern blot analysis. (DOC 48 kb) [file 12284_2016_137_MOESM1_ESM.doc]

| **Gene** | **Accession No.** | **Forward primer (5’-3’)** | **Reverse primer (5’-3’)** | **Size (bp)** |
| --- | --- | --- | --- | --- |
| OsWRKY80^a^ | Loc_Os03g6381 | CCGATGCAACGTAATGTG | GGATTATCAGGCTCCAACTC | 302 |
| OsWRKY80 | Loc_Os03g6381 | AACAGCAGCAGCAGCAGA | GTCGGCGTAGAGATTGAATGG | 92 |
| OsWRKY4 | LOC_Os03g55164.2 | CGATCCCAAGTGCGTCCTG | GGCACGGCTCATCAGCAAC | 191 |
| OsPR1a | AJ27843 | GGCACGAGTCGATCTCCA | ACCAGCAAGCAGCAGGAT | 139 |
| OsPR1b | AK107926 | GCAGGACTACGTGAGGCT | CCAGTTGCTGGAGTGGAT | 145 |
| OsPR3 | D16221 | GGCGTTCTGGTTCTGGATGAC | CGCCGTTGATGATGTTGGTC | 143 |
| OsPR5 | OSU77657 | AACTACCAGGTCGTCTTCTGC | GCGTGCGGGCTTATTCTTA | 145 |
| OsPR10/PBZ1 | D38170 | AGGACTACCTCGTCGCTCAC | GGACATTTCTGCGGCTCT | 120 |
| OsLOX | D14000 | CGGCAACTCCACTGTCCTC | CGTCTGTCCCGGCAAGTAA | 124 |
| OsAOS | AY062258 | GACTTCACCGCCCTCAAC | TGCCATAGGAGCCACAGG | 131 |
| OsPAL/ZB8 | KF556681 | CGAGTTCAACGCCGACAC | GAGCGGATACGACCTGCA | 160 |
| OsCHS | NM_001058538 | GAGAATGGGTAGCCAAGAAT | TCAAGGAGGTGATTGGAGTT | 153 |
| OsActin1 | AK071586 | CCGAGACCTCACTGACCATC | TAATCAAGGGCGACATAAGC | 123 |
| OsRUBQ1 | AF184279 | GTGGCCAGTAAGTCCTCAGC | GAAACGGGACACGACCAAGG | 116 |
| *R. solani* 28S rRNA |  | GCCTTTTCTACCTTAATTTGGCAG | GTGTGTAAATTAAGTAGACAGCAA | 137 |

**Table S1 Gene-specific primers for quantitative real-time PCR or Northern blot analysis**

a, for Northern blot
