# Supplementary material for: OsWRKY80-OsWRKY4 Module as a Positive Regulatory Circuit in Rice Resistance Against Rhizoctonia solani
Source: Rice (N Y). 2016 Nov 25;9:63. doi: 10.1186/s12284-016-0137-y (PMC5124021; doi:10.1186/s12284-016-0137-y)
Supplement: Additional file 2: Table S2. — Specific primers of transcriptional activity anlysis in yeast cells. (DOC 34 kb) [file 12284_2016_137_MOESM2_ESM.doc]

**Table S2** Specific primers of transcriptional activity anlysis

| **Name** | **Forward primer (5’-3’)^a^** | **Reverse primer (5’-3’)^b^** | **Size (bp)** |
| --- | --- | --- | --- |
| WRKY80 | GGAATTCCATATGTATGGATATGATGGAGGAGGAGG | CGGGATCCGAACTTGTGCCACTGATGATCATAG | 1162 |
| dN1 | GGAATTCCATATGATACTATCCCGCCCCCCA |  | 982 |
| dN2 | GGAATTCCATATGTCTATGGGCATGGAGGAAGTAC |  | 562 |
| dC2 | GGAATTCCATATGTATGGATATGATGGAGGAGGAG | CGGGATCCCTTGTGGCGGCTGCTGGA | 856 |
| dC1 |  | CGGGATCCTGTGATCAGTGCAGGCTGC | 904 |
| dN1C1 | GGAATTCCATATGATACTATCCCGCCCCCCA | CGGGATCCTGTGATCAGTGCAGGCTGC | 724 |

a, containing a NdeI site, underlined; b, containing a BamHI site, , underlined.
