# Supplementary material for: OsWRKY80-OsWRKY4 Module as a Positive Regulatory Circuit in Rice Resistance Against Rhizoctonia solani
Source: Rice (N Y). 2016 Nov 25;9:63. doi: 10.1186/s12284-016-0137-y (PMC5124021; doi:10.1186/s12284-016-0137-y)
Supplement: Additional file 3: Table S3. — Specific primers for amplification of the full and 5’-deleted promoters of OsWRKY80. (DOC 29 kb) [file 12284_2016_137_MOESM3_ESM.doc]

**Table S3** Specific primers for amplification of the full and 5’-deleted promoters of *OsWRKY4*

| **Name** | **Forward primer (5’-3’)^a^** | **Reverse primer (5’-3’)^b^** | **Size (bp)** |
| --- | --- | --- | --- |
| OsWRKY4-P1 | TCACTCGAGTACAAGTATTTTGAAACTGAAAA | TCCCCCGGGTTAGCTTCCTTTGCTTCT | 1466 |
| OsWRKY4-P2 | TCACTCGAG AGAAAGCTGCTGTTTATCA |  | 881 |
| OsWRKY4-P3 | TCACTCGAGCATATGTCACATCGTTGAA |  | 388 |
| OsWRKY4-P4 | TCACTCGAGTCCAATCCCAACACATCTCGC |  | 128 |

a, containing a SalI site, underlined; b, containing a SpeI site, , underlined.
